# A Comparative Analysis of Aroma Profiles of *Soju* and Other Distilled Spirits from Northeastern Asia

**DOI:** 10.3390/foods13213368

**Published:** 2024-10-23

**Authors:** In-Seo Hwang, Chan-Woo Kim, Bo Ram Kim, Bo-Ra Lim, Ji-Ho Choi

**Affiliations:** Fermented Food Science Division, National Institute of Agricultural Sciences, RDA, Wanju 55365, Republic of Korea; his0753@korea.kr (I.-S.H.); kcw5142@korea.kr (C.-W.K.); kinj56@korea.kr (B.R.K.); dlaqhfk548@korea.kr (B.-R.L.)

**Keywords:** *soju*, *baijiu*, *shochu*, aroma characteristics, raw materials, fermentation starter, distillation

## Abstract

The *soju* (Korean traditional distilled liquor) market is increasing worldwide. However, in contrast to well-explored distilled liquors, including *baijiu* (China) and *shochu* (Japan), *soju* is less investigated, with limited research on its aroma characteristics. To facilitate better understanding of the aroma characteristics of *soju*, this study aims to overview recent research on the flavor characteristics of *soju* and compare data with those of *baijiu* and *shochu*, well-established products in the market. *Soju* is generally made using rice and *nuruk* (a traditional Korean fermentation starter). Previous studies have reflected that the aroma characteristics vary with raw materials’ nutrition percentages, microbial taxa influenced in fermentation starters, and/or pressure reduction during distillation. The research on the aroma characteristics of *baijiu*, characterized by solid-state fermentation involving *qu* (a traditional Chinese fermentation starter), is focused on differences in regional characteristics of the flavor type. Research on the aroma characteristics of *shochu* has primarily demonstrated that the microbial community could contribute significantly to the development of specific aromatic compounds and/or attributes. Moreover, the association of the aroma characteristics of *baijiu* and *shochu* with their volatile compound development by the determination of selective ingredients has been examined. Understanding the current research progress can potentially facilitate the improvement in the aroma characteristics of *soju*.

## 1. Introduction

*Soju* (Korea), *baijiu* (China), and *shochu* (Japan) are the representative types of distilled liquor used in Northeast Asia. Historically, Korea, China, and Japan have been clustered in the same cultural region associated with various cross-cultural characteristics, including dietary culture and food technology. Distilled liquor is characterized by a clear appearance and a high alcohol content resulting from distillation. Its distinctive aroma depends on the characteristics of the ingredients and the distillation conditions. Various distilled liquors are available globally; however, this review focuses on the Northeast Asian distilled liquors *soju*, *baijiu*, and *shochu*, with special emphasis on their aroma characteristics.

In Korea, *soju* refers to both distilled and diluted liquors, but the production process of diluted *soju* was imported owing to liquor taxation laws during the Japanese colonial period [1]. The market strategy report of alcoholic beverages in Korea indicates the dominance of diluted *soju* in the domestic market [2]. In 2021, the market share of diluted *soju* amounted to USD 3096 million (40.1% of the total liquor market), with beer (41.0%) representing the majority of total sales with diluted *soju*, whereas traditional liquors represented only 1.1%. However, the share of traditional liquors has steadily increased with the recently increased popularity of traditional foods; an increase from KRW 40.0 to 94.2 billion (0.43% to 1.07% of the total) was noted, and distilled *soju* accounted for approximately 11.4% of all traditional liquors, with the share increasing from KRW 33,161 to 64,628 million (2017–2021) with an increased number of distilled *soju* manufacturing licenses (from 64 to 152 companies) [2].

As of 2003, the total liquor production in China amounted to 32 million tons, with *baijiu* accounting for 10.3% (CNY 55 billion); however, exports of *baijiu* were extremely low compared to the domestic market. *Baijiu* is classified depending on the fermentation process and/or the use of various raw materials. The trend of the *baijiu* market has changed according to the needs of younger consumers preferring cheaper *baijiu*; this led to changes in demand patterns in the liquor market, previously dominated by high-priced *baijiu* [3].

The *shochu* market accounts for USD 4.7 billion, representing 9.3% of the total liquor market (2019). Similar to the trend in China, most sales occur domestically. The sales of *shochu* have significantly expanded its consumption. It is characteristically consumed by mixing it with water or other beverages, leveraging its high alcohol content. Using a wide variety of raw materials, research on the flavor characteristics of *shochu* has been conducted extensively, leading to a better understanding of flavor characteristics, including the development of its flavor wheel [4,5].

Although the distilled *soju* market has seen significant growth, the limited understanding of its aroma characteristics hinders its further development compared to that of *baijiu* or *shochu*. The objective of this review was to analyze recent research on the aroma characteristics of distilled *soju*, which can potentially facilitate a better understanding of the characteristics of *baijiu* and *shochu*.

## 2. Previous Research on Aroma Characteristics of Distilled Liquor in Northeast Countries

Research on the flavor characteristics, including sensory science and instrumental analysis, of *soju*, *baijiu*, and *shochu* has been widely conducted in several countries. In Korea, most studies included instrumental analysis of *soju* reflecting aroma compound profiles using gas chromatography/mass spectrometry; Table 1 lists the previously identified volatile organic compounds (VOCs) of *soju*. Most previous studies focused on rice-based *soju*, which is potentially attributable to the prevalence of *soju* derived from rice. The VOC profile of *soju* mainly indicates the presence of alcohols, acids, ketones, and a large proportion of esters. Additionally, VOC profiles differ with various raw materials and/or distillation methods. Furthermore, several studies have focused on the development of sensory lexicons in *soju* using descriptive sensory analyses to understand the flavor characteristics of *soju* (Table 2). The commonly identified aroma attributes include sweet, alcohol, fruit, sour, and yeast, whereas flavors and tastes frequently include alcohol, fruit, bitter, sweet, and sour.

Previous findings based on other Northeast Asian countries are summarized in Table 3. In China, research on the flavor characteristics of *baijiu* primarily focused on the manifestation of regional flavor characteristics; however, in Japan, most studies determined the aroma characteristics with various types of raw materials, as *baijiu* is categorized based on its flavor characteristics, which regionally vary owing to the variable fermentation procedures and/or starter cultures, while *shochu* is classified based on the type of raw material used. Moreover, research on the flavor characteristics of *baijiu* and *shochu* revealed the influence of fermentation conditions and yeast strains on the increase in specific VOCs that affect the overall quality of the spirit.

Some research groups have comparatively analyzed flavor characteristics with various raw materials used to produce *soju*; however, most have focused on determining the differences in VOC profiles induced by the different fermentation starters (Table 4). The knowledge of correlations between the VOC profiles and sensory flavor characteristics of distilled *soju*, as well as of the microfermentation and flavoromics-based mechanisms underlying VOC generation that influence the quality of *soju*, remains limited.

## 3. Aroma-Characteristic-Related Factors Involved in the Liquor Production Procedure

### 3.1. Raw Materials and Processing Methods

In Northeast Asia, the production of distilled liquor generally involves the fermentation of starchy grains by inoculating them using natural fermentation agents or a specific yeast strain. Saccharification of raw materials converts raw starch into an alpha-starch structure, which facilitates enzymatic breakdown, including saccharification and alcohol fermentation upon inoculation with the yeast strain [15,45]. Although similar methods are followed to produce grain-based distilled liquor in Northeast Asian countries, some significant differences induce regional differences in aroma characteristics.

#### 3.1.1. Distilled Soju

**Figure 1:**
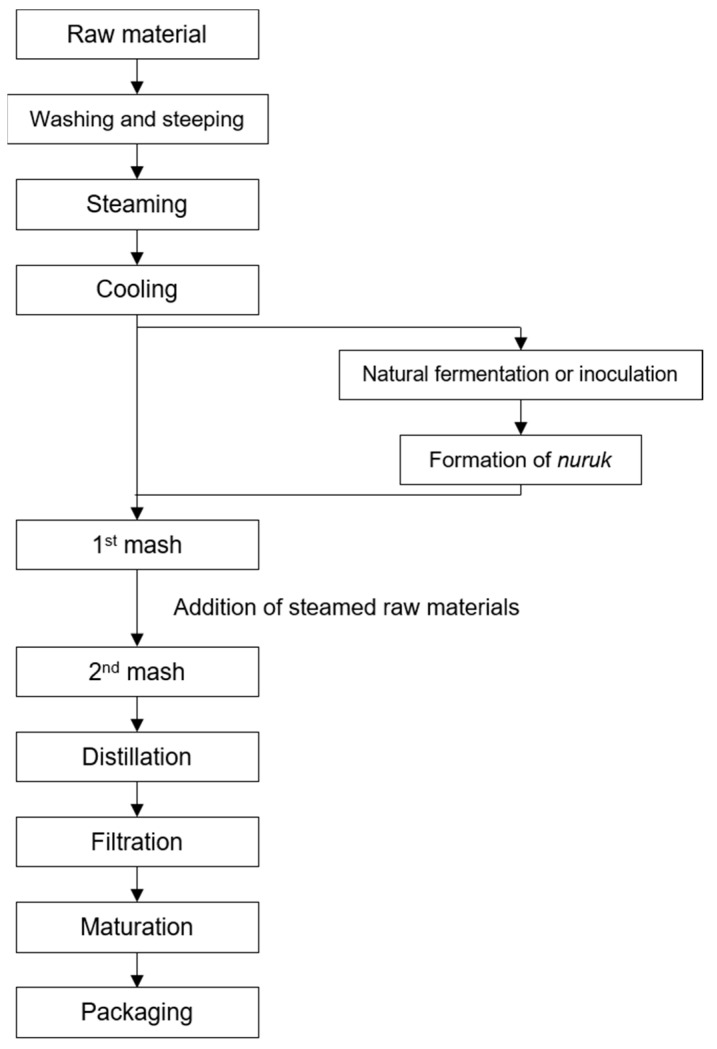
The general production process of *soju* in Korea.

In Korea, traditional *soju* is primarily produced from rice; however, the use of barley, wheat, and sweet potatoes has been reported. Wheat is rarely used to produce *soju* owing to its higher status as an edible grain compared to rice in the Korean Peninsula’s cultivation system. According to Korean liquor tax laws, *soju* is distilled using fermented starch-based materials (except in the continuous distillation process), e.g., grains; a fermentation starter (*Guk*); and water. Although the fermentation procedure of *soju* varies slightly in terms of fermentation period, temperature, and ingredient ratios, both the first and secondary mashes are typically produced using atmospheric pressure or vacuum distillation processes (Figure 1).

The characteristic aroma profiles of distilled *soju* produced using different raw materials were analyzed using instrumental and sensory analyses (Table 4). Woo et al. (2023) and Chin et al. (2024) determined that major VOCs of rice-derived distilled *soju* mainly comprise alcohol, esters, and aldehydes, which include various ethyl esters, acetaldehyde, methanol, furfural, acetic acid, N-propanol, isobutanol, n-butanol, isoamyl alcohol, 2-phenyl ethyl acetate, and 2-phenyl ethanol [7,46]. Woo et al. (2023) and Lee et al. (2012) reported several aromas of rice-derived distilled *soju*, which include alcohol, acetone, sour, sweet, wine, fruit, yeast, and oak; moreover, various flavors/(after)tastes include sweetness, sourness, bitterness, alcohol, tingling, astringency, cooling sensation, swallowing, and body [16,46].

A few studies on *soju* reported main ingredients other than rice; however, these reports demonstrated some differences in aroma characteristics. Hong et al. (2020) reported higher concentrations of some esters, including isoamyl acetate, ethyl octanoate, and phenylethyl acetate, as well as higher barley aroma and yeast flavor, detected through descriptive sensory analysis, in barley-derived *soju* than in rice-derived distilled *soju* [14]. Jeong and Seo (2012) determined the VOC profile of distilled *soju* produced via different distillation methods using potatoes and revealed the prevalence of esters and alcohols [9]. Furthermore, several studies have elucidated the differences in flavor characteristics based on the degree of polishing and/or cultivar of the raw materials. Kim et al. (2015) and Park et al. (2010) reported that the expression of VOCs in sweet potato-derived *soju* varies depending on the cultivar; decanoic acid and ethyl dodecanoate were more prominently expressed in the Hobak cultivar than in the Jinhongmi cultivar, while similar levels of other VOCs were detected. They also noted significant differences in the expression of monoterpene alcohols between sweet potato cultivars [10,47]. Furthermore, the brewing characteristics of rice cultivars and the correlation between the fermentation characteristics of fermented beverages and the degree of rice polishing (*Makgeolli*) have been explored [48,49,50]; however, to the best of our knowledge, no studies have focused on flavor characteristics.

#### 3.1.2. Baijiu

**Figure 2:**
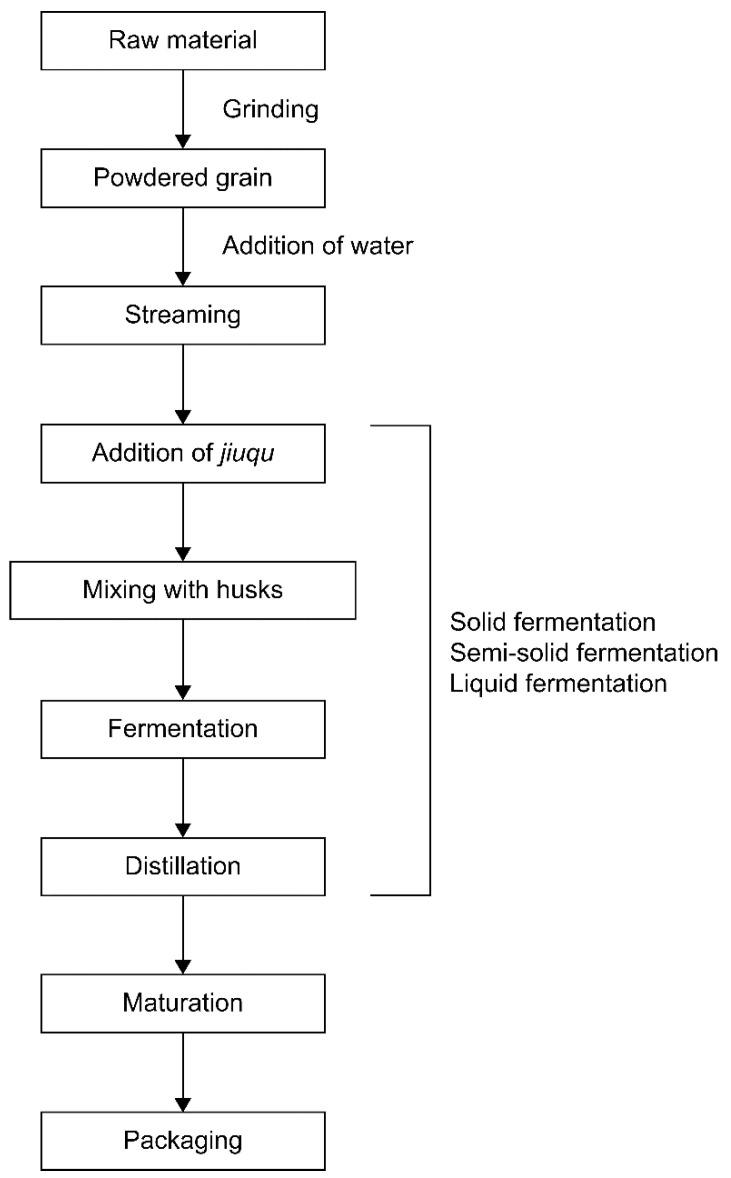
The general production process for *baijiu* in China.

The characteristics of *baijiu* vary greatly depending on the raw materials and fermentation methods used [51]; various grains (sorghum, wheat, corn, rice, and glutinous rice) are used as the raw materials. Figure 2 presents the general procedure for producing *baijiu*. The *baijiu* production method distinctively involves solid-state fermentation [51,52]. Solid fermentation differs from the general fermentation methods used in other Northeast Asian countries [52]. In the case of strong-aroma type *baijiu*, the most common type of *baijiu*, fermentation typically lasts for 30–90 days, during which saccharification and fermentation occur simultaneously [53]. It involves the use of *qu* (fermentation starters), and most raw materials remain solid throughout the fermentation and distillation stages [54]. In this strategy, lower temperatures are maintained during the steaming of raw ingredients and saccharification compared to other fermentation methods, which encourages the involvement of various microbes in fermentation. Liquid and liquid–solid combination fermentation methods have also been adopted for producing *baijiu* which are distinguished by the use of the main ingredient of mash and/or *qu* [54]. Moreover, these various types of fermentation methods influence flavor; *baijiu* is classified based on flavor characteristics, such as strong aroma, light aroma, sauce aroma, rice aroma, and mixed aroma induced by different production methods, the selection of fermentation starter, and/or fermentation regions [55,56].

The primary factor that influences the differences in the flavor characteristics of *baijiu* from other grain-based distilled liquors is considered to be its solid-state fermentation. Most research related to the production method of *baijiu* focus on the expression of aromatic compounds depending on the fermentation state [55,57]. Zhao et al. (2021) reported that the solid-state saccharification stage in the production of rice-flavor *baijiu* not only produces early alcohol that inhibits the contamination of mash but also reduces the number of short-chain fatty acids compared to the liquid-state saccharification method. This could be related to the generation of ester compounds that can contribute to the cheesy aromas of distilled liquor, together with the higher alcohol production in solid-state fermentation due to the higher protease activity [57]. However, previous studies examining the volatile compound profiles of *soju* and *shochu* also reported large quantities of various alcohols in their results as well as ester compounds (Table 1) [22,26,58]. Given that a variety of grains besides rice are used for the production of *baijiu* and *shochu*, no significant relationship between the fermentation state and formation of volatile compound profiles is observed; other factors contributing to the production of liquor may also influence the observed flavor characteristics.

#### 3.1.3. Shochu

**Figure 3:**
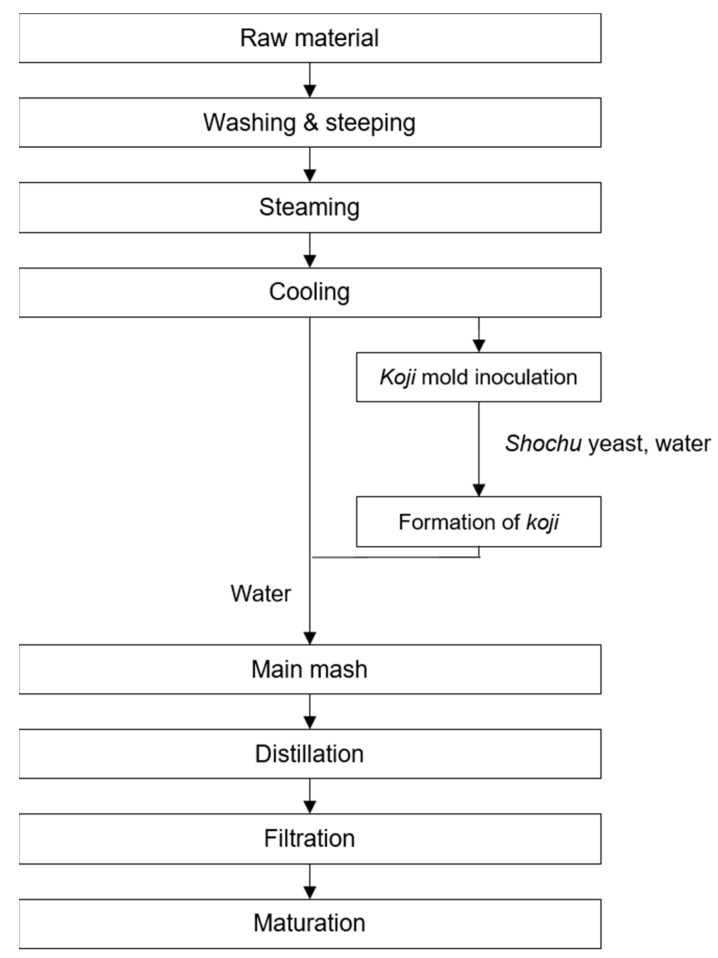
The general production process for *shochu* in Japan.

Figure 3 illustrates the general production process of *shochu* in Japan. *Shochu* is made from a wide variety of raw materials, including sweet potato, barley, rice, buckwheat, brown sugar, corn, and sake lees, while *awamori* refers to a specific type of *shochu* made in Okinawa Prefecture using restricted types of rice and *koji* fungus [59,60]. Similar to *soju*, based on distillation conditions, *shochu* is divided into diluted and distilled *shochu* by Japanese tax regulations [60].

The raw-material-associated variation in the aroma characteristics of *shochu* has been extensively investigated. Rice *shochu* is the most common type of this liquor; studies of rice *shochu* are mainly focused on the development of particular VOCs related to flavor quality [17,18,19]. Ethyl lactate and ethyl caproate, which contribute to the aroma characteristics of *shochu*, are considered major VOCs. Therefore, extensive research has been conducted on the production of these compounds in rice *shochu* under various fermentation conditions, using selective strains and production methods to enhance their formation [17,18]. Osafune et al. (2022) reported the differences in 2-furanmethanethiol development between barley-derived *shochu* and *awamori*; the production of 2-furanmethanethiol in barley *shochu* by atmospheric distillation is a potentially critical factor contributing to the generation of the characteristic roasted aroma of barley *shochu* [19]. Many raw materials are adopted for making *shochu*; various types of *shochu* contain different major VOCs; for instance, linalool and α-terpineol in sweet potato *shochu* [21], pyrazines and furans, β-damascenone, and guaiacol for sugarcane *shochu* [22] represent the VOCs of corresponding types of *shochu*. Moreover, Okutsu et al. (2016) demonstrated the association of the cultivation period of sweet potato with different concentrations of monoterpene alcohols, β-damascenone, rose oxide, and fatty acid esters in *shochu*, which is potentially attributable to the changes in physiochemical properties during cultivation [61]. Okutsu et al. (2023) reported that different amounts of amino acids in different cultivars affect levels of VOCs derived through the Maillard reaction (pyrazines and furans) in the final product [22]. As such, research trends of the flavor characteristic differences in *shochu* based on its raw ingredients are focused on the expression of specific aroma-active compounds. While dynamic variation in the aroma characteristics of *soju* with its raw materials is scarce because *soju* is predominantly produced with rice, broader diversity of aroma characteristics was observed in *shochu* due to its use of various ingredients such as sweet potatoes and barley.

Additionally, while studies in Korea (*soju*) mostly conducted the identification of volatile compound profiles and/or the increase/decrease in certain volatile compounds, research on *shochu* takes a more targeted approach to the formation mechanism of ingredient-specific, odor-active compounds which could impact flavor expression (Table 3). Because the overall production processes of *soju* and rice *shochu* share similar procedures for manufacturing, it is considered that the differences in aroma characteristics between *soju* and *shochu* are more likely influenced by the selection of ingredients and/or fermentation starters rather than production methods.

### 3.2. Fermentation Starters

The main difference between Western and Eastern liquors is the use of granule-based starters during fermentation, particularly in Korea, China, and Japan. In Western countries, fermentation generally involves a saccharification process to obtain soluble sugars before alcohol fermentation using endogenous enzymes expressed through grain germination, especially with malt. However, in Eastern countries, particularly Northeast Asian countries, fermentation starters comprising naturally or artificially inoculated fungi in granular forms are cultured to produce enzymes via the generation of microorganisms. The composition of microorganisms in the fermentation starters varies according to the procedure and/or raw materials used. Microorganism enzymes are involved in starch degradation while simultaneously facilitating alcohol fermentation. Consequently, these fermentation starters can significantly regulate the aroma characteristics of each distilled liquor.

#### 3.2.1. Nuruk

*Nuruk* is prepared using various grains, such as wheat, barley, rice, and sweet potatoes, either alone or in combination with raw materials. Depending on its form, *nuruk* is mainly divided into wadded (*Byeonggok*) and scattered, spread-out (*Sangok*) forms and further varies based on its shape and size. The production of traditional *nuruk* involves natural fermentation, hosting a diverse microbial community, including various species of *Saccharomyces*, *Pinchia*, *Aspergillus*, *Rhizopus*, *Mucor*, *Lactobacillus*, and *Leuconostoc*. Yu et al. (1996, 1998) reported that the microbial composition of traditional *nuruk* can vary; 97 species of molds, 47 species of yeast, and 19 bacterial species have been reported to be involved [62,63], with *Bacillus subtilis* and *Lactobacillus casei* being the most common bacteria. Moreover, among lactic acid bacteria, *Leuconostoc mesenteroides* is commonly detected. Traditional *nuruk* can induce diversity in the sensory and physiochemical characteristics of the final products based on its production region/fermentation conditions (temperature and humidity), yet the reproducibility of the products is relatively low. In addition, the detection of high amounts of bacteria suggests an uncontrolled manufacturing system; therefore, the use of modified *nuruk* has been recently introduced to produce alcoholic beverages [64]. Modified *nuruk* generally involves only a single microbial inoculation; primarily, *Aspergillus oryzae* and *Aspergillus luchuensis* are used to adjust the brewing characteristics of the final products. 

Because *soju* is generally made with rice, studies on the development of diverse aroma characteristics of *soju* tended to focus on the impact of the fermentation starter. As the production of *soju* has become industrialized, there is still insufficient quality characteristic information about the use of traditional *nuruk* in the final product, including aroma characteristics, due to heavy usage of *koji* in alcoholic fermentation and the natural fermentation method of traditional *nuruk* [65]. Therefore, modified *nuruk* has been developed to address these limitations. This shift is reflected in the research trends; most studies have focused on differences in the characteristics of distilled *soju* prepared using traditional and modified *nuruk*. Synthesis of VOCs, including alcohol, carbonyls, acids, esters, sulfur-containing compounds, and N-containing compounds, was significantly related to microorganisms in the fermentation starter profile, especially esters and fusel oils in the final *soju* products [38]. Lee et al. (2014) reported significant differences in the levels of several acids and alcohols; octanoic acid potentially related to the off-flavor of *soju* is more abundant in modified *nuruk*-based *soju*, whereas traditional *nuruk soju* showed higher levels of acetic acid (sour aromatics) and phenylethyl alcohol than the modified *nuruk*-based *soju* [38]. Moon and Cheong (2018) reported that traditional *nuruk* produced lower amounts of fusel oils and esters compared to modified *nuruk* inoculated with *Aspergillus oryzae* [41]. Several studies have isolated selected yeast strains to enhance the aromatic characteristics of traditional *nuruk*. *Saccharomyces cerevisiae* N4 and N9, which have high acid resistance, were isolated from traditional *nuruk* and induced higher alcohol production than commercial yeast, with differences in the development of volatile compounds, including i-BuOH, isobutanal diethyl acetal, ethyl caprate, and tetradecanoic acid [39].

#### 3.2.2. Qu

*Qu* is a fermentation starter used in China that is widely used for the production of alcohol and various other fermented products. *Jiuqu* is a type of *qu* used in alcohol production. *Jiuqu* is primarily derived from barley, wheat, peas, cereal flour, and/or bran with variations and categorized as *Daqu*, *Xiaoqu*, *Fuqu*, and mixed *qu* based on usage and production methods/raw materials [45]. Similar to *nuruk*, *qu* undergoes natural fermentation involving microbes, including *Sporolactobacillus*, *Clostridium*, *Mycobacterium*, *Flavobacterium*, *Candida*, *Pichia*, *Issatchenkia*, *Aspergillus*, and *Penicillium* [66]. *Daqu* also serves as a raw material for liquor production, significantly affecting the aroma; in *baijiu* production, large proportions of *Daqu* are used, whereas *Xiaoqu*, made from rice, contains fewer types of microorganisms than *Daqu*, resulting in relatively lighter aromas. These two types of *qu* are sometimes mixed. *Fuqu* is produced by artificially inoculating *Aspergillus* on fermented bran to create various aroma profiles; however, the aroma characteristics are still unbalanced, necessitating further investigation [54].

As the selection of *qu* is highly related to its flavor, the aroma characteristics of *qu* are selected considering their influence on final products. Zhang et al. (2012) identified that strong-flavor *Daqu* contained N-containing compounds, alcohols, and phenolic volatile compounds, including a significant level of hexanal (green), phenylacetaldehyde (floral, rose), and 4-ethyl guaiacol (clove, unbalanced) [67]. 

Moreover, regional characteristics with flavoromics relationships were determined by identifying major VOCs in several types of *baijiu* [32,68]. While recent research of *soju* and *shochu* tended to focus on modification of the microbial community in fermentation starters and their contribution to changes in flavor characteristics, studies of *baijiu* were characterized by highlighting the influence of regional differences, noting that the the aroma profiles of specific types of *baijiu* and/or *qu* were influenced by regional characteristics. Recent studies were conducted on the variation in microbial composition in *qu* with regional differences and dominant microbial communities, including environmental factors related to the development of volatile compounds. A study on sauce-flavor *baijiu* identified 20 region-specific volatile compounds, although the descriptive sensory analysis results showed no significant differences in flavor across regions [32]. Together, regional variations in flavor profiles of strong-flavor *baijiu* were identified as being influenced by different patterns of microbial communities, especially fungal diversity, which is attributed to climatic differences across China [35].

This regional diversity of *baijiu* is considered to be more pronounced due to the geological characteristics of China compared to Korea or Japan. In Korea, while traditional *soju* varieties such as Andong *soju*, *Igangju*, and *Munbaeju* are considered regionally distinct types of distilled liquors, only their historical and cultural significance has been highlighted, with no critical emphasis on the regional factors affecting the generation of aroma characteristics. Similarly, in Japan, while Ryukyu’s awamori—made from local rice and black *koji* (*Asp. luchuensis*)—is recognized as a regional product, there has been no notable focus on the impact of regional characteristics on the aroma profile diversity.

#### 3.2.3. Koji

*Koji* is generally prepared from rice, barley, or sweet potatoes using a single inoculation step. The fermentation processes in Korea and China commonly involve the use of various molds; contrastingly, the types of *koji* specifically depend on the single microorganism used as the inoculum: yellow *koji* (*Aspergillus oryzae*) is widely used in various fermented foods and alcoholic beverages. White *koji* (*Aspergillus luchuensis* mut. kawachii) and black *koji* (*Aspergillus luchuensis*) are solely used for *shochu* production. *Aspergillus luchuensis* produces citric acid at the early stages of fermentation, which prevents the growth of other bacteria and enhances the safety of the product. Although *Aspergillus niger* produces citric acid, its aroma characteristics are undesirable, leading to its exclusion from use [60].

Research of the aroma characteristics of *koji* mainly focused on the effect of *koji* on the final product of enzymatic synthesis by the microbial community in VOC production. Yuan et al. (2023) determined the effects of *koji* on the aroma characteristics of rice *shochu* and the differences in yeast strains related to the production of VOCs, and the use of *Aspergillus oryzae* for *koji* was reported to elevate alcohols and sweet-related free fatty acids with a less sour flavor in rice *koji* compared to other strains [26]. Moreover, dimethyl trisulfide and hexanal in *koji* can induce the synthesis of esters related to fruity aroma, and the formation of isovaleraldehyde, ethyl caprylate, ethyl caproate, and ethyl 2-methylbutyrate can influence specific aroma characteristics of rice *shochu* [58]. Research on the enhancement of specific VOCs by adjusting the microbial community profile in *shochu* mash fermentation was widely reported. Similar to other liquor-making procedures, the production of volatile compounds in *shochu* is related to the selected yeast strain [69]. Additionally, Yuan et al. (2015) and Tan et al. (2016) demonstrated the enhancement of ethyl caproate and ethyl lactate, which are related to the enhancement of the sensory quality of rice *shochu*, using a caproic acid-producing bacterial consortium and lactic acid bacteria [17,18].

Determination of the relationship between the microbial community of *koji* and development of VOC in *shochu* is highly influenced by the production method of *koji*—a single inoculation of a microbial strain. Both *nuruk* and *qu* contain complex microbial communities due to the naturally derived fermentation procedure, and the specific microorganisms have been identified to have a significant effect on the generation of volatile compounds and/or aroma characteristics.

The use of *koji* in *soju* production is also abundantly observed in the research field. Unlike studies in Japan, which were more focused on the flavor control with microfermentation for the expression of specific volatile compounds, studies of *soju* production often adopted *koji* as a control method. Studies have been conducted to determine the changes in volatile compound profiles in *soju* depending on vacuum/atmospheric distillation and the use of modified *nuruk* [36] and to identify quality changes in *soju* made from traditional/modified *nuruk* over various aging periods, employing *koji* in the production of *soju* as a control method [70].

### 3.3. Distillation Conditions

Distillation is a significant regulatory factor that influences the aroma characteristics of distilled liquor owing to the various odor-active VOCs produced by thermal reactions at different temperatures [1,71]. Aroma characteristic differences based on distillation condition were identified to have a major impact in *soju*. Atmospheric pressure distillation involves hot steam at 80–90 °C and the generation of VOCs in *soju* with atmospheric distillation, which become more varied with the increase in distillation time. Furthermore, several aldehydes, such as ethyl acetate, isoamyl acetate, ethyl alcohol, fusel oils, and higher fatty acid esters (ethyl palmitate and ethyl linoleate) are produced in large quantities in the primary stage; however, their levels decrease during distillation [1]. Several characteristics, including acid value, acetic acid, and β-phenyl alcohol, tend to become more pronounced as distillation progresses, and higher mineral contents and furfural level are expressed. The sensory characteristics of *soju* with atmospheric distillation included a mild taste with stronger characteristics due to these thermally induced secondary organic compounds [43,71].

**Figure 4:**
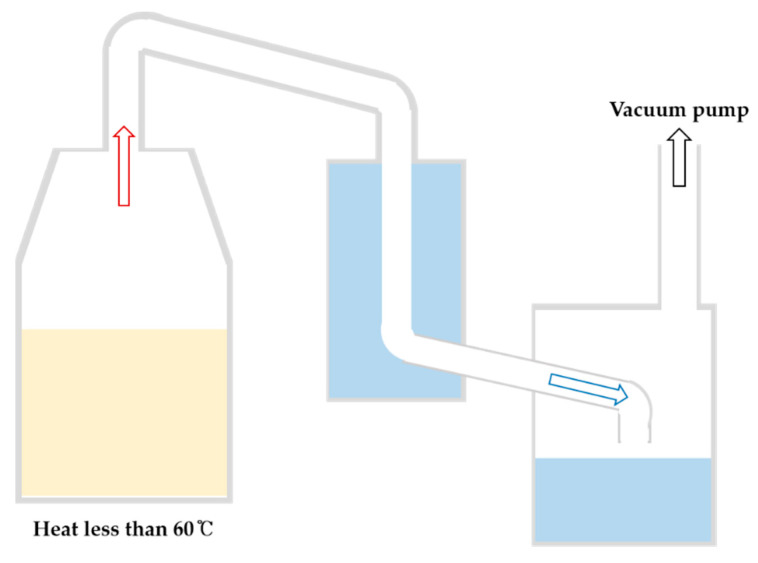
The general schematic diagram of the vacuum distillation of liquor.

Vacuum distillation allows indirect heating while maintaining low-pressure conditions to reduce the boiling point (Figure 4) [1,71], and the VOCs originating from heat are not expressed; therefore, the different VOC profiles can induce a characteristic aroma profile different from that of *soju* produced by atmospheric distillation. In particular, VOCs with low boiling points are released during the early to middle stages of distillation. A slight difference was identified between vacuum and atmospheric distillation because vacuum distilled compounds are mostly derived from fermentation rather than heat-induced changes [43,71]. However, several studies have demonstrated that VOCs with medium to high boiling points, which were released during the late stages of distillation, were more abundant in *soju* subjected to atmospheric distillation than in *soju* subjected to vacuum distillation, and isoamyl alcohol (whisky) and decanoic acid (sweet, nutty) were more abundant in *soju* subjected to atmospheric distillation than in *soju* subjected to vacuum distillation; this difference was attributed to the high boiling points of the compounds [38]. Consistent with previous reports, the 2-thiobarbituric acid value, a marker of oxidative deterioration, was observed, particularly in the later stages of atmospheric distillation process [72]. Moreover, furfural sharply increased in the late stage of distillation in atmospheric distillation, which is not detected in vacuum distillation. This is attributed to the thermally induced carbohydrate degradation during atmospheric distillation [12,43]. However, acid-related organic compounds show minimal differences between the two methods and are present in exceptionally lower quantities in vacuum-distilled *soju* because acetaldehyde is released in the early stages of distillation as a gas through the vacuum pump [1].

## 4. Conclusions

This review elucidates the recent research progress on the flavor characteristics of *baijiu*, *shochu*, and distilled spirits with cultural and technical similarities to *soju*, which potentially enhances the understanding of the aroma characteristics of *soju*. 

Research trends on the factors influencing the development of flavor characteristics during the production of *shochu* has focused more on the identification of distinctive flavor characteristics differing according to the selection of main ingredients and on increasing the production of flavor compounds that positively affect the final product’s sensory qualities by the formation of a selective microbial community during *koji* production. In the case of *baijiu*, studies have mainly focused on identifying the flavor intensity and its characteristics based on the different types of the mash during fermentation, while studies on *qu* have focused on identifying the diversity of microbial communities across different regions. Although various studies of *soju* have been conducted on the differences in flavor characteristics according to raw materials and on identifying the microbial communities of *nuruk* as a fermentation starter, limited studies have been conducted on connecting these findings to the final product’s flavor characteristics. Unlike the research strategies of *shochu* and *baijiu*, the final products of which have diverse quality based on flavor profiles, studies of *soju* have not yet explored how current findings can be applied to the development of the final products. Moreover, research in the area of microfermentation aimed at improving the quality characteristics of *soju* is limited, together with the understanding of the development of aroma characteristics during fermentation. This review provides insights for future research on the flavor characteristics of *soju*.

## Figures and Tables

**Table 1:** VOCs previously identified in distilled *soju*.

Compound	Rice		Potato		Sweet Potato	Odor Threshold (mg/kg) [6]	
	Atmospheric	Vacuum	Atmospheric	Vacuum	Atmospheric	Water	Ethanol/Water Solution
Alcohols							
1-Propanol	O	O	O	O		8.5056	830
Ethanol	O	O	O	O		950	120
1-Butanol	O	O				0.4592	820
Isobutyl alcohol	O	O	O	O		6.5052	40
Isoamyl alcohol	O	O	O	O		0.004	56.1
Isohexanol	O					0.82–4.1	50
1-Hexanol		O				0.0056	8
n-Octanol				O		0.1258	10
Nonanol				O		0.0455	
Nonen-1-ol				O		0.13	
2-Methyl-1-butanol		O				0.0159	32
3,7-Dimethyl-6-octen-1-ol		O				0.062–2.2	0.1
3-Methylthio-1-propanol	O	O				0.12323	0.5
Benzyl alcohol	O	O				2.54621	159
Phenylethyl alcohol	O	O				0.56423	10
Undecanol					O	0.086–0.41	
1-Dodecanol					O	0.016	1
2-Ethyl-1-hexanol		O				25.4822	
Hexadecanol					O		>1.1
Acids							
Acetic acid	O	O	O	O		99	26
Butanoic acid		O				2.4	10
4-Hydroxybutanoic acid	O						
Propanoic acid		O				2.19	830
Aldehydes							
Furfural	O	O				9.562	15
5-Methyl furfural		O				1.11	16
Benzaldehyde		O				0.75089	5
Benzeneacetaldehyde	O				O	0.0063	0.001
Hexdecanal							
1-Tetradecanal					O		
n-Valeraldehyde			O			0.012	0.11
Acetaldehyde		O		O		0.0251	10
Hexanal				O		0.005	0.07–0.1
2,4-Nonadienal		O				0.0001	0.0026
Esters							
Ethyl formate		O				8.9–89	
Ethyl acetate	O	O	O	O		0.005	7.5
n-Propyl acetate	O	O				2	65
Isobutyl acetate	O	O				0.025	3.4
Phenylethyl acetate	O	O				0.25	0.25
Ethyl butanoate		O				0.0009	0.02
Diethyl butanedioate		O					100
Ethyl butyrate	O	O				0.009	0.02
Isoamyl acetate	O	O		O		0.00015	0.03
Isoamyl propanoate		O				0.0086–0.043	
Ethyl valerate		O				0.0058	
Ethyl lactate		O				50–250	100
Dodecyl acetate				O			
Ethyl caproate	O	O				0.0022	0.005
Methyl caprate				O		0.07	
Ethyl 2-hydroxyisocaproate		O					
Ethyl heptanoate	O	O			O	0.0019	0.3
Isobutyl hexanoate	O	O		O			
Ethyl caprylate	O	O	O	O		0.0193	0.002
Isoamyl hexanoate	O	O				0.32	1.4
Propyl octanoate	O	O					
Butyl octanoate		O			O		
Ethyl 9-decenoate		O		O			
Ethyl nonanoate	O	O		O		0.377	
Isobutyl caprylate	O	O			O		
Ethyl caprate	O	O	O	O		0.005	0.51
Isoamyl caprylate	O	O		O		0.07	0.6
Propyl decanoate	O	O		O			
Isobutyl decanoate	O	O			O		
Ethyl benzeneacetate		O					
Methyl salicylate	O	O				0.04	0.071
Ethyl laurate	O	O	O	O	O	5.9	0.64
Isoamyl decanoate	O	O		O	O		>5.0
Ethyl myristate	O	O		O		4	494
Isoamyl laurate	O				O		100
Ethyl palmitate	O	O	O	O		2	>14
Ethyl palmitoleate	O			O	O		10
3-Methylbutyl hexanoate		O					
3-Methylbutyl octanoate		O					
Methyl 2-hydroxybenzoate		O			O		
Ethyl benzeneacetate		O					
Ethyl 3-methylbutyl butanedioate		O					
Methyl hexadecanoate		O			O	>2	
Ethyl-(E)-11-hexadecenoate					O		
Ethyl octadecanoate	O				O		>0.5
Ethyl oleate	O				O		0.87
Ethyl linoleate					O		0.45
Propyl lactate	O			O	O		
Butyl lactate				O			10
Diethyl phthalate			O				
Bis(2-ethylhezyl)hexanedioate					O		
Ethyl-4-decanoate					O		
Ethyl pentadecanoate	O				O		
Diethyl butanedoate					O		
2-Hydroxy-methyl benzoate					O		
Ethyl-3-heptenoate					O		
3-Phenylethyl-2-propenoate					O		
Ethyl-3-methylbutyl pentadecanoate					O		
Ethyl undecanoate		O					
2-Methyl decanoate					O	0.0043–0.0088	
Ethyl 2-furoate		O					1
Ketones							
2-Nonanone		O				0.041–0.082	
Acetophenone	O					0.065	
2-Dodecanone					O	0.042–0.083	
Butyrolactone		O				>1	100
Trans-Whiskey lactone		O					
Oaklactone		O					
Acetals							
1-(1-Ethoxyethoxy)pentane	O	O					
Others							
1-Ethyl-2,3-dimethylbenzene					O		
1-Ethyl-3,5-dimethylbenzene					O		
1,2,4,5-Tetramethylbenzene		O					
2,6-Dimethyl-2,6-octadiene					O		
1-Cyclohexylheptene					O		
2-Pentadecyle-1,3-dicxolane					O		
Phenol		O				58.58525	7.1
Eugenol		O			O	0.00071	0.005
2-Methoxy phenol		O				0.00084	0.03
Benzofuran		O					
2-Acetylfuran		O				15.0252	
2-Methylbenzofuran		O					
2,4-Dimethylheptane		O					
4-Methyloctane		O					

References [7,8,9,10,11,12]. VOC, volatile organic compound.

**Table 2:** Sensory lexicon identified from distilled *soju* in previous studies.

Sensory Attributes		References	Sensory Attributes		References
*Aroma*	Brandy	[13,14]	*Flavor/taste*	Alcohol	[13,14,15,16]
	Sweet	[13,14,15,16]		Bitter	[13,14,15,16]
	Acetone	[13,14,16]		Sweet	[13,14,15,16]
	Alcohol	[13,14,15,16]		Fruit	[13,14,15]
	Fruity	[13,14,15,16]		Sour	[13,14,15,16]
	Gusu	[13]		Salty	[15]
	Sour	[14,15,16]		Mint	[15]
	Wine	[16]		Barley	[15]
	Yeast	[14,15,16]		Earthy	[15]
	Oak	[16]		Metal	[15]
	Earthy/woody	[15]		Yeast	[14]
	*Nuruk*	[14]	*Texture*	Body	[13,14,15,16]
	Sauce-like	[15]		Swallow	[13,14,15,16]
	Green grape	[15]		Persistence	[13,14]
	Pineapple	[15]		Pungent	[14,16]
	Barley	[14,15]		Astringent	[14,15,16]
	Metal	[15]		Cooling sensation	[14,15,16]
	Bleach	[14,15]		Tingling	[16]
				Spicy	[15]

**Table 3:** Previously reported aroma characteristics of *baijiu* and *shochu*.

Liquor	Raw Materials	Type	Objectives	Major Results	References
*Shochu*	Rice	*Shochu*	VOC enhancement	Ethyl caproate, ethyl lactate	[17]
			VOC synthesis	Ethyl caproate	[18]
		Barley *shochu*, *awamori*	Flavoromics focusing on roasted aroma	2-Furanmethanethiol	[19]
	Rice, sweet potato	*Imo*-*shochu*	Fermentation temperature control for sensory quality	Sweet/acidic taste	[20]
	Sweet potato	Sweet potato *shochu*	Flavor characteristic determination	Linalool, α-terpineol	[21]
	Sugarcane	Sugarcane *shochu*	Aroma profile differences by cultivar	MRPs (pyrazines and furans), β-damascenone, guaiacol	[22]
			Liming effect determination	pH-relevant volatiles	[23]
	Barley	Barley *shochu*	Aroma characteristic difference in *koji*	Ethyl lactate, 3-methyl-1-pentanol, ethyl benzoate, diethyl succinate, citronellol, 2-phenyl acetate	[24]
		*Shochu*, *awamori*	Major VOC threshold measurement		[25]
	Rice	*Shochu*	Effect of *koji* VOC on final product	Amino acids, volatile compounds, sensory lexicon	[26]
*Baijiu*	Rice	Rice-flavored *baijiu*	VOC profile	Key compound—ethyl lactate	[27]
			Major VOC threshold measurement		[28]
			Effect of rice cultivar on VOCs	Isopentyl acetate, ethyl hexanoate, benzeneacetaldehyde, phenethyl acetate, undecane-2-one, ethyl decanoate	[29]
		Sauce-flavored *baijiu*	Regional flavor characteristic determination		[30]
			Key VOCs and aroma lexicon development		[31]
			Regional-specific aroma characteristic determination		[32]
		Strong aroma *baijiu*	Regional chemosensory characteristic determination		[33]
			Correlation between sweetness and VOCs	Ethyl hexanoate, hexyl hexanoate, ethyl 3-methylbutanoate	[34]
	*Qu*, environmental elements	Strong aroma *baijiu*	Regional flavor profile difference-inducing factor	Fungal diversity differences by climate	[35]

MRP, Maillard reaction product; VOC, volatile organic compound.

**Table 4:** Previous studies conducted on aroma characteristics of distilled *soju*.

Raw Materials	Objectives	Differences		References
Rice	VOC profile	Fermentation starter	Traditional/modified *nuruk*	[36]
		Distillation condition	Atmospheric/vacuum distillation	[36]
	Quality optimization	Commercial yeast	Domestic/commercial yeasts	[37]
	VOC profile	Fermentation starter	Traditional *nuruk*, *koji*	[38]
		Distillation condition	Atmospheric/vacuum distillation	[38]
	Liquor production properties	Yeast strain	*Saccharomyces cerevisiae* N4, N9 isolated from traditional *nuruk*	[39]
		Yeast strain	*Saccharomyces cerevisiae* 88-4 isolated from traditional *nuruk*	[40]
		Fermentation starter	*Koji* (*Aspergillus kawachii*, *Aspergillus oryzae*), traditional/modified *nuruk*	[41]
	VOC profile	Fermentation starter	*Koji* (*Aspergillus luchuensis*), traditional *nuruk*, crude amyloytic enzyme	[42]
	VOCs, sensory characteristics	Yeast strain	*Saccharomyces cerevisiae* 88-4 and GNIA2 (selective mutant) isolated from traditional *nuruk*	[7]
	Liquor production properties	Distillation condition	Atmospheric/vacuum distillation	[7]
			Atmospheric/vacuum single distillation, atmospheric continuous distillation	[43]
		Organic acid addition	Acetic, citric, lactic acid (0.3% *w*/*v*)	[44]
Sweet potato	Liquor production properties	Cultivar	Jinhongmi, Hobak	[10]
		Fermentation starter	*Koji* (*Aspergillus awamori* Nakazawa, *Aspergillus kawachii*, *Aspergillus oryzae*), modified *nuruk*	[10]
	VOCs, sensory characteristics	Cultivar	Yeonmi, Jeungmi, Shincheonmi, Shinwyeulmi	[10]

VOC, volatile organic compound.

## Data Availability

No new data were created or analyzed in this study. Data sharing is not applicable to this article.
